# Twelve-Month-Old Infants Anticipatorily Plan Their Actions According to Expected Object Weight in a Novel Motor Context

**DOI:** 10.3389/fpubh.2015.00032

**Published:** 2015-02-23

**Authors:** Michaela Boone Upshaw, Jessica A. Sommerville

**Affiliations:** ^1^Early Childhood Cognition Lab, Department of Psychology, Center for Child and Family Wellbeing, University of Washington, Seattle, WA, USA

**Keywords:** infancy, weight perception, action planning, motor inference, motor adaptation

## Abstract

Planning actions in anticipation of object weight is fundamental to skilled action production. The present study investigated whether infants can apply weight information gained from direct actions on objects in order to plan their actions according to object weight in a novel and indirect motor context. In the present study, two groups of 12-month-old infants were provided with experience acting directly on two blocks of different weights and colors (70 versus 470 g; red versus yellow). Subsequently, infants were administered a novel task in which the same blocks (standard condition; *n* = 60), or blocks of the reversed color–weight pairings (switch condition; *n* = 60), were placed out-of-reach, on top of a cloth, and infants were encouraged to retrieve the block by acting on the cloth. Infants in the switch condition produced more failed cloth pulls when retrieving the 470 g block, due to inadequate generation of anticipatory force, relative to infants in the standard condition. This demonstrates that infants’ force on the cloth was prospectively generated based on their mental representation of the supported block’s weight, which was formed through their previous direct actions on the object. Thus, infants use information about the weight of an object in order to anticipate how to obtain that object in a novel and indirect problem-solving context.

## Introduction

The ability to successfully navigate the physical world depends critically on our knowledge and understanding of a wide range of object properties. Chief among these properties is object weight: representing and understanding the consequences of weight is central to our ability to plan actions on objects, understand other people’s behavior, and predict event outcomes. Imagine helping a friend move to a new apartment: accurately representing object weight allows one to decide when to use a single hand versus two hands in order to lift a packed box, to recognize that when a box slips through one’s friend’s grasp it is likely because she has underestimated the weight of the box, and to understand that a box packed full of books, but not a box packed full of pillows, can serve to prop open an apartment door. Given the centrality of weight perception and the importance of understanding the impact of object weight on others’ actions and event outcomes, it is perhaps unsurprising that the rudiments of weight perception can be traced back to infancy. Infants can discriminate objects on the basis of weight [e.g., Ref. (1)], adjust their actions on objects according to weight (2), and use information about the outcome of physical events (e.g., the degree to which an object compresses a supporting object) in order to determine the weight of an object (3).

In addition to differentiating objects on the basis of object weight and adjusting actions online based on object weight, a critical component of weight perception involves generating actions in *anticipation* of an object’s weight. Adults use their prior experience with objects (4, 5) as well as visual cues that are typically associated with weight [e.g., size; (6, 7)] in order to anticipatorily scale the force of their actions according to object weight. For example, adults generate greater lifting force when lifting an object that is anticipated to be heavy than one that is anticipated to be light (4). Evidence suggests that the origins of this ability can be traced back to infancy. After previous experience interacting directly with objects of varying weight, infants aged 9 months and older exert more force when lifting an object they expect to be heavy than an object they expect to be light (8, 9).

The novel question addressed in the current study is whether infants can use information about the weight of an object garnered through their direct actions in order to anticipatorily plan their actions according to the object’s weight in a novel and indirect motor context. Past work provides some evidence for anticipatory action planning in infancy. For example, infants pre-configure their hand shape in order to conform to the size, shape, and orientation of an object prior to object contact (10–13), and the kinematics of infants’ reaches toward objects, such as the speed of the reach, vary as a function of what infants intend to do with the object once they pick it up (14). However, no existing work has investigated whether infants’ ability to use representations of object weight extends to planning their actions as a function of object weight in a novel and indirect motor context.

The present study investigated whether 12-month-old infants would encode object weight information acquired through directly lifting an object and subsequently apply this information to scale the force of their actions on an intermediary object that supported the previously lifted object. We selected 12-month-old infants for this experiment because this is the age at which infants initially become highly successful at solving means-end tasks [i.e., tasks that require the infant to act on an intermediary object in order to obtain a goal object; (15)]. Infants first received a training phase in which they were encouraged to repeatedly lift two plastic blocks that were identical in size, shape, and material, but that varied in weight (70 versus 470 g) and in color (red versus yellow). Accordingly, one block was of standard weight for its size and material (70 g) whereas the other block was decidedly heavier (470 g). After the training phase, infants received a novel cloth-pulling task in which a single block was placed on top of a piece of cloth, such that the block itself was out of the infants’ reach but the cloth was not. Thus, in order to retrieve the block infants must first act on the cloth. Infants in the standard condition were administered the cloth-pulling task using the same blocks that they interacted with during the training phase. In contrast, infants in the switch condition were administered the cloth-pulling task using blocks that were visually identical to those used in the training phase, but were actually of the reversed color–weight pairings, unbeknownst to infants (i.e., if the red block weighed 70 g during the training phase, it weighed 470 g during the cloth-pulling task).

Our goal was to investigate whether infants anticipatorily varied the force of their actions on the cloth based on their representation of the supported block’s weight. We focused on instances in which infants attempted to pull the cloth in order to retrieve the 470 g block, yet, in doing so, failed to bring the block toward them, due to an under-application of force on the cloth (“failed cloth pulls”). We hypothesized that if infants accurately encoded the 470 g block’s weight during the training phase, and then used this information to plan their actions on the cloth, infants in the switch condition should produce more failed cloth pulls when retrieving the 470 g block than infants in the standard condition. In other words, infants in the switch condition (but not infants in the standard condition) should under-represent the weight of the 470 g block during the cloth-pulling task (i.e., represent the block weight as 70 g), due to the surreptitious reversal of the blocks’ color–weight pairings after the training phase; consequently, when pulling on the cloth, infants in the switch condition should anticipatorily generate force that is insufficient for retrieving the 470 g block, leading to failed cloth pulls.

Thus, the present study was designed to investigate whether infants would use their representation of the block’s weight in order to guide their actions in a novel and indirect context, at the initial point at which infants were presented with the problem and in the absence of trial-and-error learning. A demonstration that infants use experience gained from directly acting on an object in order to guide their actions in a novel and indirect problem-solving context would provide evidence that infants form “motor inferences.” That is, it would demonstrate that infants use information gained from prior experience in order to generate a novel action or motor plan that guides behavior in a new context.

## Materials and Methods

### Participants

One hundred and twenty healthy, full-term, 12-month-old infants (*M* = 12 months, 16 days; range = 12 months, 2 days–13 months, 21 days; 59 females and 61 males) participated in the study. Infants were recruited from a large city in the Pacific Northwest and were primarily Caucasian (*n* = 82; Asian: *n* = 5; Black: *n* = 1; Hispanic: *n* = 1; Multiracial: *n* = 21; and Other-race: *n* = 3; ethnicity data was not provided for *n* = 7 participants). Parents provided written informed consent before the testing procedures, and all study procedures were approved by the University’s Internal Review Board before the research was conducted. Twenty-six additional infants were tested but excluded from the final sample due to fussiness (*n* = 14), experimental error (*n* = 6), not interacting with the blocks during the training phase (*n* = 4), or failing to solve the cloth-pulling task during both pre-test trials (*n* = 2). Infants were randomly assigned to the standard condition (*n* = 60; *M* = 12 months, 17 days; 31 males), or the switch condition (*n* = 60; *M* = 12 months, 15 days; 30 males).

Participants sat in a high chair. In cases of excessive distraction or fussiness, infants were moved to their parents’ lap (standard condition, *n* = 11; switch condition, *n* = 13). The study was conducted on a 61 cm × 91.4 cm × 76.2 cm wooden table with an attached sliding top, which allowed the experimenter to arrange the stimuli out of the infant’s reach before starting each trial.

### Procedure

Each infant took part in a training phase, followed by two cloth-pulling pre-test trials, and four cloth-pulling test trials. Infants were seated directly in front of the testing table throughout the experiment. The experimenter sat to the right of the infant, facing the adjacent side of the table, approximately 51 cm from the infant.

#### Training phase

The training phase was designed to allow the infant to directly interact with each block in order to discover and encode each block’s respective weight properties. Infants were given two plastic blocks, one red and one yellow, each of different weights, one 70 g and one 470 g (color–weight pairing counterbalanced across infants). Each block measured 8.9 cm on each side. The 470 g block was weighted by inserting metal washers and cotton batting (included to eliminate noise from the washers) into the ordinarily hollow interior of the block and was sewn back together using plastic fishing wire. The 70 g block was not weighted, but was similarly stitched, in order to maintain an identical appearance (aside from color) to the 470 g block.

The training phase started with a free play period, in which the experimenter placed both the 70 and 470 g blocks in front of the infant, and allowed the infant to freely explore and manipulate them. The experimenter encouraged the infant to spend equivalent time interacting with each block, by directing infants’ attention as appropriate, during the first 40 s of the training phase (i.e., free play period). During the next part of the training phase, the experimenter modeled an action with a single block, and the infant was encouraged to reproduce the experimenter’s action. For example, the experimenter lifted and placed each block (separately, and one at a time) on top of an inverted plastic container (25.4 cm × 17.8 cm × 10.2 cm) that served as a platform, and infants were encouraged to reproduce the experimenter’s lifting and placing action. Then, the experimenter lifted and dropped each block (separately, and one at a time) into a clear, plastic bucket (17.1 cm × 14.6 cm × 12.7 cm), and infants were again encouraged to reproduce the experimenter’s lifting and dropping action. Each of these actions was modeled twice, and infants were encouraged to reproduce each action twice, with both the 70 and 470 g blocks (in alternation; order counterbalanced across infants).

#### Pre-test trials

The goal of the pre-test trials was to determine that infants were capable of solving the cloth-pulling task, before administration of the test trials; given prior work, we anticipated that 12-month-old infants would readily solve this problem (15). Two pre-test trials were administered with novel bath toys, which did not resemble the blocks used in the training phase. To administer the pre-test trials, the experimenter moved the sliding table top out of the infant’s reach, laid a rectangular cloth (38.1 cm × 20.3 cm) on the table, and placed a bath toy (e.g., a multi-colored spaceship or a pink bug) on the far end of the cloth. The experimenter then tapped the toy, while saying, “*Can you get it? Can you get it?*” in order to encourage infants to retrieve the bath toy.

#### Test trials

After the pre-test trials, infants were administered two test trials with each block, for a total of four test trials.

For infants in the standard condition, the blocks used in the test trials were the same blocks used during the training phase (i.e., if the red block was 470 g during the training phase, it weighed 470 g during the test trials). For infants in the switch condition, the color–weight pairings were reversed from the training phase (i.e., if the red block was 70 g during training, it weighed 470 g during test trials). Test trials were administered using the same procedure as the pre-test trials: the experimenter moved the sliding table top out of the infant’s reach, laid the cloth on the table, and placed either the 70 g or the 470 g block on the far end of the cloth (order counterbalanced across infants; block weight alternated on each test trial). The experimenter then tapped on the block while saying, “*Can you get it? Can you get it?*” before sliding the table top within the infant’s reach, initiating the test trial. If the infant made no attempt to retrieve the block within 10 s, the experimenter encouraged the infant to retrieve it again by tapping on the block and saying, “*Can you get it? Can you get it?*” Infants were given ten additional seconds to retrieve the block. If, after a period of 20 s, the infant still had not retrieved the block, the experimenter placed the block directly in front of the infant. Each infant was given several seconds at the end of every test trial to interact with the block before starting the next test trial, in order to encourage the infant’s behavior and maintain his/her attention.

Importantly, while handling the blocks during the training phase and the test trials, the experimenter always lifted each block with a single hand, and controlled the pace and measure of her arm movements, so that no visual clues to block weight were provided to the infant.

### Coding and reliability

All sessions were video recorded, and coding was completed off-line by observers who were unaware of the weight of the blocks and of the conditions in which infants participated.

#### Training phase

First, we coded time spent in simultaneous hand-and-eye contact with each block (referred to hereafter as “contact time”), in order to ascertain that infants had equal opportunity to interact with and encode the weights of both blocks. Second, we coded the number of one-handed and two-handed lifts performed with each block, in order to determine that infants adjusted their lifting actions with each block based on its weight. Lifts were operationalized as manual actions that elevated at least one corner of the block off of the table as this demonstrated that the infant had used his or her own force to vertically displace the block.

#### Pre-test trials

In order to ensure that infants were able to solve the cloth-pulling task, we coded whether infants successfully moved the bath toy within reach through their actions on the cloth for each of the two pre-test trials. Infants who failed to solve the cloth-pulling task during both pre-test trials were excluded from subsequent analysis (*n* = 2; see Participants).

#### Test trials

The primary goal of the study was to determine whether infants varied the force of their actions on the cloth as a function of their expectation of the supported block’s weight, which was based on their training experience. Accordingly, we coded the number of failed cloth pulls produced by infants when attempting to retrieve each block. Failed cloth pulls occurred whenever infants attempted to pull the cloth in order to retrieve the supported block, but failed due to an under-application of force. Failed cloth pulls were formally operationalized as instances in which the infant’s hand contacted the cloth and moved backward, yet failed to move the block *any distance whatsoever*. Furthermore, to ensure that infants’ cloth-pulling actions were clearly directed toward obtaining the block (rather than directed toward the cloth itself), failed cloth pulls were only coded and analyzed if they appeared to be planfully and intentionally directed toward the goal of retrieving the block, using criteria established by previous work (15); i.e., visual fixation on the block prior to reaching for and while pulling on the cloth; please see https://sites.google.com/site/infantsactionplanningbyweight/ for a video example of a failed cloth pull.

#### Reliability

A second observer independently coded the number of failed cloth pulls for a randomly selected 25% of participants. Reliability was high across all four test trials: first 470 g block test trial, *K* = 0.84, inter-rater agreement = 90%; first 70 g block test trial, *K* = 1.0, inter-rater agreement = 100%; second 470 g block test trial, *K* = 0.77, inter-rater agreement = 83.3%; second 70 g block test trial, *K* = 1.0, inter-rater agreement = 100%.

## Results

### Training phase

#### Contact time

Paired samples *t*-tests confirm that infants spent equal time interacting with the 470 g block (standard condition: *M* = 34.65 s, *SE* = 1.57 s; switch condition: *M* = 36.35 s, *SE* = 1.53 s) and with the 70 g block (standard condition: *M* = 34.12 s, *SE* = 1.76 s; switch condition: *M* = 36.22 s, *SE* = 1.91 s) within each condition: standard condition: *t*(59) = 0.41, *p* = 0.69; switch condition: *t*(59) = 0.07, *p* = 0.95. Importantly, infants’ contact time with the 470 and 70 g blocks did not vary as a function of condition, as confirmed by independent samples *t*-tests: 470 g block: *t*(118) = 0.77, *p* = 0.44; 70 g block: *t*(118) = 0.81, *p* = 0.42.

#### Lifts

**Figure 1:**
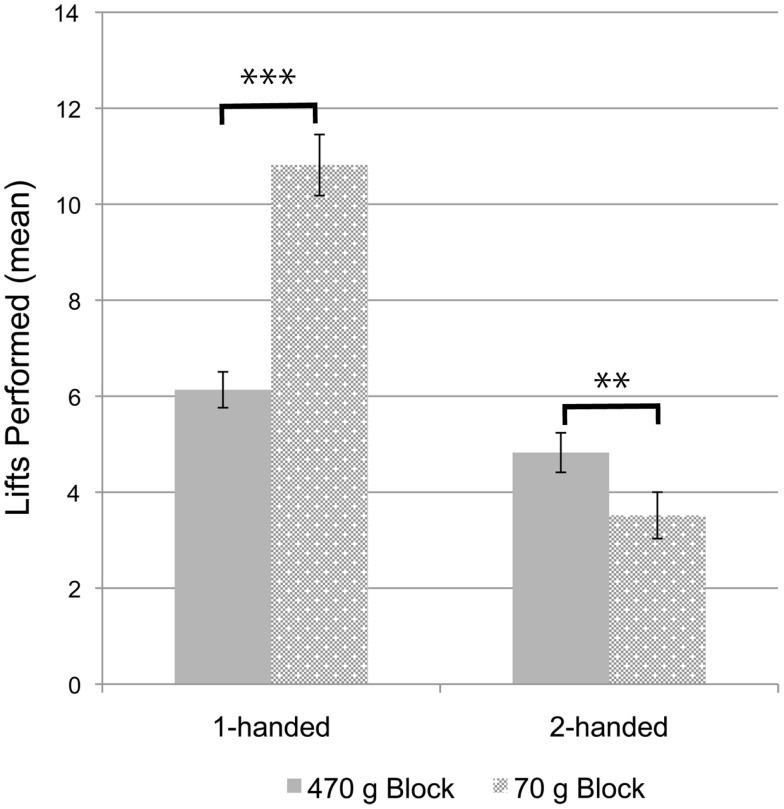
**Mean number of lifts performed with each block during the training phase as function of the number of hands used (collapsed across conditions)**. Error bars represent *SE*. **p = 0.001; ***p < 0.001.

To address whether infants successfully encoded the blocks’ respective weights during training, we analyzed one- versus two-handed lifts performed with each block (see Table 1 and Figure 1). Looking at the sample as a whole, paired samples *t*-tests confirm that infants performed more one-handed lifts with the 70 g block than with the 470 g block, *t*(119) = 8.27, *p* < 0.001, *d* = 0.77, and more two-handed lifts with the 470 g block than with the 70 g block, *t*(119) = −3.31, *p* = 0.001, *d* = 0.27. These findings provide evidence that infants adapted their actions on the basis of block weight, and, accordingly, that infants encoded the respective weights of the blocks during the training phase. Importantly, independent samples *t*-tests confirm that the number of block lifts performed during the training phase did not differ between conditions: one-handed lifts of the 70 g block, *t*(118) = 0.13, *p* = 0.90; two-handed lifts of the 70 g block, *t*(118) = 0.55, *p* = 0.58; one-handed lifts of the 470 g block, *t*(118) = 1.07, *p* = 0.29; two-handed lifts of the 470 g block, *t*(118) = 1.36, *p* = 0.18; and the total number of one- and two-handed lifts with both blocks, *t*(118) = 1.18, *p* = 0.24.

**Table 1:** **Means (and *SEs*) for the number of one- and two-handed lifts performed during the training phase**.

Variable	Standard condition	Switch condition
One-handed lifts
70 g block	10.73 (0.72)	10.90 (1.06)
470 g block	5.73 (0.42)	6.53 (0.62)
Two-handed lifts
70 g block	3.25 (0.67)	3.78 (0.70)
470 g block	4.27 (0.48)	5.38 (0.67)

### Pre-test trials

#### Solve rates

On average, infants were successful on 1.9 (*SE* = 0.03) out of two pre-test trials. An independent samples *t*-test confirms that performance on the pre-test trials did not differ by condition, *t*(118) = 0.60, *p* = 0.55, suggesting that infants in both conditions were equally and highly skilled at solving the cloth-pulling task.

### Test trials

#### Failed cloth pulls

In order to determine if performance on the cloth-pulling task varied by condition, test trial pair, or block weight, we conducted a 2 × 2 × 2 repeated measures ANOVA on the number of failed cloth pulls performed on each of the four test trials. Block weight (70 versus 470 g) and trial pair (first pair versus second pair) were within-subjects variables, and condition (standard versus switch) was the between-subjects variable. We hypothesized that infants in the switch condition would produce more failed cloth pulls on the first 470 g block test trial than infants in the standard condition, that is, before infants in the switch condition were aware of the color–weight pairing reversal.

**Figure 2:**
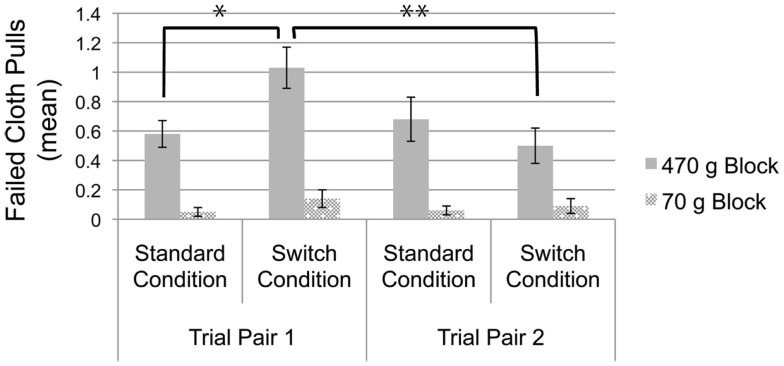
**Mean number of failed cloth pulls when attempting to retrieve each block, presented as a function of trial pair and condition**. Error bars represent *SE*. *p = 0.008; **p = 0.001.

We found a main effect of block weight, *F*(1, 91) = 63.13, *p* < 0.001, $\eta_{p}^{2}$ = 0.41. A follow-up paired samples *t*-test confirms that, overall, infants performed more failed cloth pulls on the 470 g block test trials (*M* = 0.73, *SE* = 0.08) than on the 70 g block test trials (*M* = 0.10, *SE* = 0.03), *t*(118) = 7.84, *p* < 0.001, *d* = 1.0. We also found a significant trial pair by condition interaction, *F*(1, 91) = 7.34, *p* = 0.008, $\eta_{p}^{2}$ = 0.075. Follow-up, independent samples *t*-tests reveal that performance in each condition varied as a function of trial pair: infants in the switch condition performed more failed cloth pulls than infants in the standard condition on the first pair of test trials, *t*(110) = 2.83, *p* = 0.006, *d* = 0.14, but not on the second pair of test trials, *t*(94) = −1.10, *p* = 0.27. Critically, these effects were underscored by a hypothesized three-way interaction between block weight, trial pair, and condition, *F*(1, 91) = 5.52, *p* = 0.021, $\eta_{p}^{2}$ = 0.057. Planned independent samples *t*-tests reveal that infants in the switch condition performed more failed cloth pulls on the first 470 g block test trial than infants in the standard condition, *t*(115) = 2.71, *p* = 0.008, *d* = 0.49; however, failed cloth pulls did not differ by condition on the first 70 g block test trial, *t*(111) = 1.23, *p* = 0.22, the second 70 g block test trial, *t*(104) = 0.65, *p* = 0.52, nor on the second 470 g block test trial, *t*(101) = 0.94, *p* = 0.35 (see Table 2 and Figure 2). *Post hoc* paired samples *t*-tests reveal that infants in the switch condition reduced the number of failed cloth pulls from the first 470 g block test trial to the second 470 g block test trial, *t*(47) = 3.58, *p* = 0.001, *d* = 0.50, whereas infants in the standard condition did not, *t*(51) = −0.62, *p* = 0.54.

**Table 2:** **Means (and *SEs*) for the number of failed cloth pulls performed during the test trials**.

Variable	Standard condition	Switch condition
Failed cloth pulls
First trial pair
70 g block	0.05 (0.03)	0.14 (0.06)
470 g block	0.58 (0.09)	1.03 (0.14)
Second trial pair
70 g block	0.06 (0.03)	0.09 (0.05)
470 g block	0.68 (0.15)	0.50 (0.12)

## Discussion

The primary goal of the current study was to investigate whether 12-month-old infants would apply object weight information gained through directly interacting with two blocks of the same size and shape but of different weights, in order to anticipatorily plan their actions according to object weight in a novel and indirect motor context. Specifically, following a training phase, during which infants were encouraged to act directly on the blocks (e.g., lift, drop, and freely manipulate them), infants received test trials in which they were encouraged to retrieve the same blocks (standard condition), or visually identical blocks of reversed color–weight pairings (switch condition), when the blocks were placed out-of-reach, on top of a piece of cloth. We investigated whether infants varied the force of their actions on the cloth on the basis of their mental representation of the supported block’s weight, which was established during training.

The fact that infants preferentially used one hand to lift the 70 g block and two hands to lift the 470 g block during the training phase, and did so at equal rates across conditions, provides evidence that infants encoded the respective block weights and adapted their actions accordingly. Thus, we investigated infants’ frequency of failed cloth pulls (i.e., instances in which infants pulled the cloth but failed to move the block due to an under-application of force) and compared the frequency of failed cloth pulls across the two conditions.

Infants across both conditions produced more failed cloth pulls when the supported block weighed 470 versus 70 g. It is possible that this finding emerged because infants have come to expect that objects of this size and material weigh significantly less than 470 g, based on their everyday experience with visually similar objects; a non-mutually exclusive possibility for this result is that infants are generally conservative in their use of force. In either case, the critical question of interest was whether infants’ production of failed cloth pulls would vary as a function of condition on the 470 g block test trials. Our results demonstrated that infants in the switch condition produced more failed cloth pulls on the first 470 g block test trial than infants in the standard condition. This finding suggests that infants encoded the color–weight pairings of the blocks during the training phase and used this information to anticipatorily plan their actions on the cloth according to their expectations of the supported block’s weight.

Critically, the design of our experiment allows us to rule out the possibility that differences in the number of failed cloth pulls across conditions reflects infants’ inability to adequately *adapt* their actions on the basis of online sensorimotor feedback. First, the test trials were administered in an identical manner across conditions: infants acted on the same cloth, in order to retrieve a block of identical weight. The fact that the physical properties of the task were identical across conditions, but that infants’ mental representations of the block’s weight differed, ensures that the difference in failed cloth pulls on the first 470 g block test trial across conditions was due to differences in infants’ use of force in *anticipation* of the 470 g block’s weight. The results from the training phase and pre-test trials also suggest that the results were not attributable to one condition having received more experience with the blocks during the training phase: across both conditions, infants spent equivalent time in contact with each block, and both conditions adjusted their behavior during the training phase based on the blocks’ respective weight properties. In addition, these results are not attributable to one condition being better able to solve the cloth-pulling task, as infants in both conditions solved the pre-test trials at equivalently high rates. Lastly, these findings cannot be explained by a general tendency for infants in the switch condition to underutilize force when acting on the cloth, as infants in both conditions were equally likely to produce failed cloth pulls on the second 470 g block test trial, and both conditions produced equivalent numbers of failed cloth pulls on the 70 g block test trials.

Interestingly, differences between the two conditions were found only on the first 470 g block test trial and not on the second 470 g block test trial. Infants in the switch condition significantly improved their performance between the first and second 470 g block test trials, decreasing the number of failed cloth pulls on the second 470 g block test trial relative to the first. In contrast, infants in the standard condition performed equivalent numbers of failed cloth pulls on both the first and second 470 g block test trials. This demonstrates that infants in the switch condition appropriately adjusted their behavior after discovering the color–weight pairing reversal on the first 470 g block test trial. In comparison, the standard condition did not adjust or improve their performance between the 470 g block test trials, despite having room for improvement (i.e., failed cloth pulls on the 470 g test trials exceeded those on the 70 g test trials).

An intriguing question regards *how* infants in the switch condition were able to increase their anticipatory force on the second 470 g block test trial based on sensorimotor feedback received on the first 470 g block test trial. As infants’ ability to plan their grasp in anticipation of acting on an object undergoes rapid development in the first year of life [such as matching its shape, orientation, and size; see Ref. (12, 13, 16)], we wondered whether changes in infants’ hand posture contributed to their increased generation of anticipatory force on the second 470 g block test trial. To investigate this possibility, we looked for changes in infants’ hand configuration between the first and second 470 g block test trials for a subset of infants (*n* = 40), such as whether infants grasped the cloth with the thumb and opposing fingers or pulled the cloth with a flat and open hand. However, we did not find a systematic pattern of changes between infants’ hand configuration on the first 470 g block test trial and their hand configuration on the second 470 g block test trial. This null result is similar to previous work demonstrating that infants’ ability to anticipatorily configure their grasp when acting on intermediary objects lags behind their ability to do so when acting directly on objects, and is still developing well into the second year of life (17, 18). Nevertheless, it is possible that subtle changes in infants’ hand configuration, such as their precise finger placement, contributed to their increased generation of anticipatory force on the second 470 g block test trial. However, as infants’ ability to anticipatorily generate force according to object weight is diminished when executing developmentally mature hand configurations [e.g., pincer grasp; (8, 19)], this possibility seems unlikely.

In addition, the present findings have implications for the development of the neural circuitry underlying motor planning, and force planning in particular, as well as implications for developmental disorders that are marked by deficits in such circuitry. Past research has established that the basal ganglia, a collection of sub-cortical nuclei situated in the midbrain, plays a key role in motor planning, adaptive force control, and procedural learning by trial-and-error, among other abilities (20–22). Research suggests that the basal ganglia matures earlier than most areas of the cerebral cortex and is nearly fully developed at birth (23, 24). Importantly, basal ganglia impairment has been implicated in several neurodevelopmental disorders, including developmental coordination disorder (DCD), attention deficit hyperactivity disorder (ADHD), and autism spectrum disorders (ASD). Moreover, individuals with such neurodevelopmental disorders also demonstrate a diminished capacity to plan, coordinate, and appropriately scale the force of their actions (20, 25–27).

Our results support previous research establishing early maturation of the basal ganglia by demonstrating that 12-month-old infants can anticipatorily apply force according to an object’s weight in novel contexts, and that they can rapidly adjust their behavior on the basis of sensorimotor feedback, both of which are supported in part by functioning of the basal ganglia. An interesting question for future research is whether variability in infants’ ability to anticipatorily scale force and to adapt to sensorimotor feedback in our task at 12 months of age will be related to the expression of symptoms of DCD, ADHD, and/or ASD later on in development, given prior work demonstrating deficits in scaling force in this population at older ages (28–30). As such, it foreseeable that the present task could be applied in the future as an early diagnostic marker or tool for neurodevelopmental disorders related to basal ganglia pathology.

In conclusion, this study demonstrates that a critical aspect of motor planning is already in place by the end of the first year of life: infants utilize representations of object weight gained from acting directly on objects in order to anticipatorily plan their actions in a novel and indirect motor context. This ability is a pervasive and important aspect of mature motor behavior, as we are often faced with the need to perform actions in novel situations and in which weight information plays a prominent role, such as loading a dolly when helping a friend move between apartments. Based on these and related findings (31), we suggest that infants form “motor inferences”: they can flexibly combine information and/or representations gleaned from different motor acts in order to generate novel plans of action in the absence of trial-and-error experience.

## Author Contributions

JS developed the study concept and experimental design. Testing and data collection were performed by research assistants. MU coded the data. Both authors contributed to data analysis and interpretation. MU drafted the paper and JS provided critical revisions. Both authors approved the final version of the manuscript for submission.

## Conflict of Interest Statement

The authors declare that the research was conducted in the absence of any commercial or financial relationships that could be construed as a potential conflict of interest.
